# Phylogenetic analysis of ligninolytic peroxidases: preliminary insights into the alternation of white-rot and brown-rot fungi in their lineage

**DOI:** 10.1080/21501203.2014.895784

**Published:** 2014-03-25

**Authors:** Li-Wei Zhou, Yu-Lian Wei, Yu-Cheng Dai

**Affiliations:** State Key Laboratory of Forest and Soil Ecology, Institute of Applied Ecology, Chinese Academy of Sciences, Shenyang 110164, P. R. China

**Keywords:** concerted evolution, birth-and-death evolution, selective pressure, gene structure, positive selection

## Abstract

White-rot and brown-rot fungi employ different mechanisms to degrade lignocellulose. These fungi are not monophyletic and even alternate in their common lineage. To explore the reason for this, seventy-six ligninolytic peroxidases (LPs), including 14 sequences newly identified from available basidiomycetous whole-genome and EST databases in this study, were utilized for phylogenetic and selective pressure analyses. We demonstrate that LPs were subjected to the mixed process of concerted and birth-and-death evolution. After the duplication events of original LPs, various LP types may originate from mutation events of several key residues driven by positive selection, which may change LP types and even rot types in a small fraction of wood-decaying fungi. Our findings provide preliminary insights into the cause for the alternation of the two fungal rot types within the same lineage.

## Introduction

Wood-decaying fungi could release nutrients, particularly carbon, from wood, and thus play an essential role in the function of forest ecosystems. For this reason, they attracted widespread attention of forest ecologists (Lonsdale et al. 2008) and other scientists representing different disciplines, such as fungal taxonomy (Lutzoni et al. 2004), forest pathology (Asiegbu et al. 2005), and biotechnological applications (Cohen et al. 2002). Two main rot types of wood are known, namely, white-rot and brown-rot (Worrall et al. 1997). The former is caused by white-rot fungi, which can almost totally decay lignocelluloses, including lignin, parts of cellulose, and hemicellulose. The latter decay is caused by brown-rot fungi, which only selectively remove cellulose and hemicellulose (Ryvarden 1991). White-rot and brown-rot fungi employ two completely different mechanisms to fully and partially degrade components of lignocellulose, and the most important difference between them is whether they have capacity to degrade lignin, one of the most abundant natural biopolymers. Therefore, they might have different evolutionary origins. Actually, the degradation capacity has been used as an important taxonomic character in Basidiomycota for more than 20 years (Redhead and Ginns 1985). Surprisingly, in the phylogeny of Basidiomycota, inferred from the combination of nuclear and mitochondrial small-subunit ribosomal DNA data, white-rot and brown-rot fungi are not separated from each other (Hibbett and Donoghue 2001). Moreover, brown-rot fungi have six independent origins within white-rot fungal branches and inversely the white-rot fungus, *Grifola frondosa* (Dicks.) Gray, is situated on a branch of brown-rot fungi (Hibbett and Donoghue 2001). Based on ancestral state reconstruction, Hibbett and Donoghue (2001) proposed that the cause for the bidirectional transformation between white-rot and brown-rot fungi is that the genes encoding lignin-degrading enzymes of white-rot fungi are also retained in the genomes of brown-rot fungi, and the difference is caused just by the change of gene expression patterns. This explanation, however, does not take the available data on expression systems into consideration. Moreover, the genome of *Postia placenta* (Fr.) M.J. Larsen and Lombard, a brown-rot fungus, lacks genes encoding true lignin-degrading enzymes (Martinez et al. 2009). Therefore, it is still not fully understood that how white-rot and brown-rot fungi alternate in their lineage from the view of lignin-degrading enzymes, of which two kinds are known, namely, ligninolytic peroxidases (LPs) and laccases. In this study, we focused on the LPs, because their reaction mechanism has been intensively studied (Hammel and Cullen 2008).

LPs are extracellular fungal peroxidases, belonging to Class-II of the plant/fungal/bacterial peroxidases superfamily, which also includes two other classes: Class-I, intracellular peroxidases and Class-III, extracellular plant peroxidases (Conesa et al. 2002; Passardi, Theiler, et al. 2007). All members of the three classes share the same heme moiety and are believed to be evolutionary closely related (Passardi, Bakalovic, et al. 2007; Morgenstern et al. 2008). Phylogenetic analyses of this superfamily are mainly focused on Class-I (Zámocký 2004; Passardi, Zámocký, et al. 2007), even if Passardi, Bakalovic, et al. (2007) explored the whole relationship of this superfamily. More recently, Morgenstern et al. (2008) focused on Class-II, but concentrated mainly on the description of LP diversity, and whether the various types of LPs were monophyletic or polyphyletic rather than on the molecular dynamics diversifying the types. Until now, there is no comprehensive and in-depth phylogenetic analysis of LPs, which are distinguished from other members of this superfamily by the capacity to degrade lignin. Three well-known LP types exist in white-rot fungi: lignin peroxidases (LiP), manganese peroxidases (MnP), and versatile peroxidases (VP). Various white-rot fungi can produce either one, two, or all of them. LiP was first described in the growth medium of *Phanerochaete chrysosporium* Burds (Kirk and Farrell 1987). It was characterized by the capacity to oxidize high redox potential aromatic substrates, such as non-phenolic lignin structures, for its tryptophan residue (W171 referred to as LiPA of *P. chrysosporium;* Doyle et al. 1998). Meanwhile, MnP was identified in the same species (Gold et al. 1989). In comparison to LiP, MnP is unable to oxidize high redox potential substrates for lack of the key tryptophan residue (Hammel and Cullen 2008). However, MnP can oxidize the relatively low redox potential phenolic lignin structures by the oxidation of Mn^2+^ by a manganese-binding site (E35xxxE39…D179 referred to as MnP1 of *P. chrysosporium;* Wariishi et al. 1991; Sundaramoorthy et al. 1994). Because VP is absent in the best studied ligninolytic fungus *P. chrysosporium,* it was not discovered until the late 1990s, in*Pleurotus eryngii* (DC.) Quél. (Camarero et al. 1999). VP is known for sharing the oxidation sites of both LiP and MnP. In addition to the three types, a fourth type of LPs, which has lost all or part of the key residues essential in the oxidization reactions, was first found in *Coprinopsis cinerea* (Schaeff) Redhead et al., by Baunsgaard et al. (1993). Although the tertiary structure is similar to that of the other types (Larrondo et al. 2005), it still needs to be investigated if it can efficiently degrade lignin for the lack of the key residues. We called it ‘CII’ short for ‘other class II peroxidase’ in this study following the nomenclature of PeroxiBase (Passardi, Theiler, et al. 2007). LPs often have several isozymes, such as the 10 LiPs and 5 MnPs in *P. chrysosporium* (Martinez et al. 2004; Vanden Wymelenberg et al. 2006). This may reflect the specificity of the LPs in response to the diversification of lignin (Passardi, Bakalovic, et al. 2007).

By the comparison of whole genome sequences of the phylogenetically closely related white-rot fungus *P. chrysosporium* and brown-rot fungus *Postia placenta,* Martinez et al. (2009) suggested that brown-rot fungi were shift from white-rot fungi by losing the capacity to degrade lignin, particularly by losing LPs. However, the phylogeny was constructed using only a few LPs, and their origin in white-rot fungi was not shown. It is proposed that the LPs originated from a cytochrome c peroxidase (CcP), belonging to the Class-I of the plant peroxidases superfamily, which changed and became able to degrade the novel appearing high redox potential polymer lignin (Passardi, Bakalovic, et al. 2007). However, the reason that different fungal species express different types of LPs and the evolutionary order of LPs were still unknown. Recently, Ruiz-Duenas et al. (2013) explored the diversity of LPs from 10 sequenced genomes, but all resulted from Polyporales. Therefore, these preliminary studies could not convincingly explain the evolutionary origin and the shift of white-rot and brown-rot fungi within the basidiomycetous lineage.

By searching the available basidiomycetous whole-genome and EST databases, we extended the annotated data set from PeroxiBase (Passardi, Theiler, et al. 2007) and assembled the most complete and nonredundant LPs with the information of gene structures. We explored the phylogeny of LPs and elucidated their evolutionary dynamics in both white-rot and brown-rot fungi taking into consideration both the types of LPs and fungal rot. The results presented below can provide preliminary insights into the nature of the alternation of white-rot and brown-rot fungi in their fungal lineage at the molecular level.

## Materials and methods

### Data mining

All sequence data used in this study were retrieved from PeroxiBase (Passardi, Theiler, et al. 2007), JGI (the DOE Joint Genome Institute website: http://www.jgi.doe.gov/) and NCBI (National Center for Biotechnology Information: http://www.ncbi.nlm.nih.gov/). LP amino acid sequences were obtained from PeroxiBase, the latest update on 22 September 2011 for LPs, and partial ones were completed using the corresponding genome sequences in JGI. To avoid redundancy, if there are several same LPs from various strains of one fungal species, only one LP amino acid sequence was selected for further analyses, and all incompletely processed transcripts (Macarena et al. 2005) were discarded. In addition, certain amino acid sequences were edited manually according to their genome sequences. *lp* gene sequences (including both exons and introns) were retrieved from the JGI or NCBI databases, if available. Six CcPs were selected as outgroups for the phylogenetic analysis.

To identify novel LP sequences, all available basidiomycetous genome sequences of both white-rot and brown-rot fungi in JGI and NCBI were screened using tBlastN with default settings and different LP amino acid sequences as queries. All queries with high sequence similarity produced similar hits. Exon–intron boundaries of the identified genomic sequences were recognized based on the alignments of ESTs and gDNA and the conserved intron splicing rule. The obtained coding sequences were translated into amino acid sequences using the ExPASy (Expert Protein Analysis System) translation tool (http://www.expasy.org/tools/dna.html). The conserved residues mainly in charge of redox functions were detected through sequence alignments using ClustalX 1.81 (Thompson et al. 1997) with default settings and comparisons of the tertiary structure using 3D-JIGSAW (Contreras-Moreira and Bates 2002) with previously verified LPs (reviewed by Martínez (2002)). Retrieved sequences without conserved heme-binding sites were discarded; however, those lacking key residues for oxidizing substrates, but with conserved tertiary structures, were included to make the analysis complete.

### Sequence alignments and phylogenetic analyses

SignalP 3.0 online server (Bendtsen et al. 2004) was used to predict the signal peptides (SPs) of all LPs and six CcPs using the neural network model with default settings. Two protein data sets, one is all LPs and six CcPs, the other is those without SPs, were used in subsequent phylogenetic analysis. The two data sets were aligned with ClustalX 1.81 using default settings (Gonnet series weight matrix, gap opening: 10, and gap extention: 0.2) and manually edited, respectively.

Three methods were performed for phylogenetic analyses of the two data sets. MEGA 3.1 (Kumar et al. 2004) was used to construct neighbor-joining (NJ) trees based on pair-wise deletion of gaps/missing data and p-distance matrix of amino acids model with 1000 bootstrap replicates. The maximum likelihood (ML) trees with 100 bootstrap replicates were constructed using PhyML 3.0 (Guindon and Gascuel 2003) with the best-fit evolutionary model (WAG + I + G + F) selected by ProtTest 2.4 (Abascal et al. 2005) according to the AIC criterion. The maximum parsimony (MP) trees with 1000 bootstrap replicates were constructed using PAUP* 4.0b10 (Swofford 1998). All characters have equal weight, and gaps are treated as missing.

### Gene structure analysis

For all known *lp* introns located in neither 5′ nor 3′ untranscripted region (UTR), only the gene sequences located between the translation start and stop codons were studied. All 66 full-length amino acid sequences with detailed gene structures were aligned with ClustalX 1.81 using default settings. Position and phase of introns were recognized based on the comparison of boundary exons and their corresponding amino acid sequences, and mapped onto the matrix of multiple alignments. The intron phase was set as 0, if an intron intervenes between two consecutive codons, 1 if an intron intervenes between the first and the second codon nucleotides, and 2 if an intron intervenes between the second and the third codon nucleotides, as described in Sanchez et al. (2003) and Zhou et al. (2008). To visualize the comparison of gene structures clearly, the introns were renamed using capital letters in alphabetical order corresponding to their appearing order in the above matrix, and the LPs from the same groups were grouped together manually.

### Selective pressure estimation

The CODEML program in PAML 4.2 (Yang 2007) was used to estimate the selective pressure on LPs. Following the recommendation of Anisimova et al. (2002), three pairs of site models, namely, M0 (one-ratio) vs. M3 (discrete), M1a (NearlyNeutral) vs. M2a (PositiveSelection), and M7 (beta) vs. M8 (beta and ω) (Yang et al. 2000; Wong et al. 2004), were employed to detect positive selection. The values of nonsynonymous/synonymous substitution rate ratio (ω = *d*N/*d*S) < 1, = 1, and > 1 mean purifying selection, neutral evolution, and positive selection, respectively. Therefore, only when the ω value of model M3, M2a, or M8 was higher than one, a likelihood ratio test (LRT; Nielsen and Yang 1998) was used to test the results of the above three nested models. The sites under positive selection were also identified by both naïve empirical Bayes (NEB) and Bayes empirical Bayes (BEB).

## Results

### Identification and distribution of LPs

**Figure 1.**
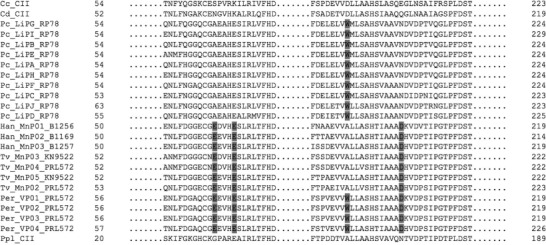
Partial sequence alignment of 24 selected LPs. Their code names are the same as those in Table 1. Dot lines mean the omitted amino acid residues. The numbers in front of and behind the residues, respectively, refer to the positions of the first and the last residues in its full-length amino acid sequence. The key residues, ExxxE…D and W, essential in the oxidization reactions are shaded gray. The LPs with ExxxE…D, W, both and neither are MnP, LiP, VP, and CII, respectively.

A total of 76 LP amino acid full-length sequences (in Supplementary File 1) were identified, including 14 novel ones, of which 4 and 10 were completed and predicted from genome sequences, respectively. The corresponding genomic data of 66 LPs were successfully retrieved. Relevant information is listed in Table 1. The 76 LPs fell in three fungal orders: Russulales, Agaricales, and Polyporales, as described by Passardi, Bakalovic, et al. (2007). Of these LPs, 20 are LiPs exclusively found in the Polyporales, 11 are VPs found in both the Agaricales and Polyporales, 37 are MnPs, and 8 are CIIs, both of which are found in all three orders. The alignment of 24 selected LPs, including CII, MnP, LiP, and VP, was presented to show the key residues essential in the oxidization reactions (Figure 1).

**Table 1.** List of LP and CcP used in this study.

Species	Entry ID (name)	Database	Code name	Type	Genomic sequence ID	Taxonomy
*Agaricus bisporus*	2402 (AbMnP01)	PeroxiBase	Ab_MnP01	MnP	AJ699058	Agaricales
*Bjerkandera adusta*	2418 (BaLiP)	PeroxiBase	Ba_LiP	LiP	E03952	Polyporales
	2307 (BaVP01)	PeroxiBase	Ba_VP01	VP	DQ060037^‡^	Polyporales
*Bjerkandera* sp.	2395 (BspVP)	PeroxiBase	Bsp_VP	VP	AY217015	Polyporales
*Ceriporiopsis subvermispora*	2377 (CsuMnP01)	PeroxiBase	Csu_MnP01	MnP	AF013257	Polyporales
	2374 (CsuMnP02a)	PeroxiBase	Csu_MnP02a	MnP	AF161078	Polyporales
	3832 (CsuMnP02b)	PeroxiBase	Csu_MnP02b	MnP	AF161584	Polyporales
	2375 (CsuMnP03)	PeroxiBase	Csu_MnP03	MnP	AF161585	Polyporales
	2376 (CsuMnP04)	PeroxiBase	Csu_MnP04	MnP	AY217670	Polyporales
*Coprinellus disseminatus*	3842 (CdCII01)	PeroxiBase	Cd_CII	CII	DQ056142	Agaricales
*Coprinopsis cinerea*	2403 (CcinCII01)	PeroxiBase	Cc_CII	CII	X70789	Agaricales
*Dichomitus squalens*	2341 (DsMnP01)	PeroxiBase	Ds_MnP01	MnP	AF157474	Polyporales
	2340 (DsMnP02)	PeroxiBase	Ds_MnP02	MnP	AF157475	Polyporales
*Ganoderma applanatum*	2405 (GapCII01)	PeroxiBase	Gap_LiP	LiP	AB035734^‡^	Polyporales
*Ganoderma australe*	3871 (GauCII01)	PeroxiBase	Gau_LiP	LiP	DQ267753	Polyporales
*Ganoderma formosanum*	3882 (GfCII01)	PeroxiBase	Gf_LiP	LiP	DQ267752	Polyporales
*Heterobasidion annosum*	3891 (HanMnP01_B1256)^*^	PeroxiBase	Han_MnP01_B1256	MnP	scaffold_9:100546-1057019	Russulales
	3881 (HanMnP02_B1169)^*^	PeroxiBase	Han_MnP02_B1169	MnP	scaffold_3:1266641-1268246	Russulales
	3844 (HanMnP03_B1257)^*^	PeroxiBase	Han_MnP03_B1257	MnP	scaffold_14:843556-845198	Russulales
	101371^†^	JGI	Han_pMnP2	MnP	scaffold_3:494513-496175	Russulales
	106090^†^	JGI	Han_pMnP	MnP	scaffold_9:1009416-1011056	Russulales
	33275^†^	JGI	Han_pCII	CII	scaffold_3:1218262-1219929	Russulales
	108376^†^	JGI	Han_pMnP5	MnP	scaffold_14:372321-374338	Russulales
*Laccaria bicolor*	191903^†^	JGI	Lb_pCII	CII	scaffold_50:308999-310856	Agaricales
*Lentinula edodes*	3886 (LedMnP01)	PeroxiBase	Led_MnP01	MnP	AB241061	Agaricales
*Phanerochaete chrysosporium*	2412 (PcLiPA_RP78)	PeroxiBase	Pc_LiPA_RP78	LiP	scaffold_19:373851-375408	Polyporales
	2413 (PcLiPB_RP78)	PeroxiBase	Pc_LiPB_RP78	LiP	scaffold_19:378294-376752	Polyporales
	6835 (PcLiPC_RP78)	PeroxiBase	Pc_LiPC_RP78	LiP	scaffold_19:394751-393151	Polyporales
	6832 (PcLiPD_RP78)	PeroxiBase	Pc_LiPD_RP78	LiP	scaffold_19:1416506-1418221	Polyporales
	2409 (PcLiPE_RP78)	PeroxiBase	Pc_LiPE_RP78	LiP	scaffold_19:360433-358710	Polyporales
	6834 (PcLiPF_RP78)	PeroxiBase	Pc_LiPF_RP78	LiP	scaffold_9:1444920-1446516	Polyporales
	2481 (PcLiPG_RP78)	PeroxiBase	Pc_LiPG_RP78	LiP	scaffold_19:448641-450192	Polyporales
	6833 (PcLiPI_RP78)	PeroxiBase	Pc_LiPI_RP78	LiP	scaffold_19:443404-441862	Polyporales
	2417 (PcLiPJ_RP78)	PeroxiBase	Pc_LiPJ_RP78	LiP	AF140062	Polyporales
	6836 (PcLiPH_RP78)	PeroxiBase	Pc_LiPH_RP78	LiP	scaffold_19:451627-454927	Polyporales
	2332 (PcCII01)	PeroxiBase	Pc_CII_RP78	CII	scaffold_10:1250082-1251701	Polyporales
	3829 (PcMnP01b)^§^	PeroxiBase	Pc_MnP01	MnP	scaffold_15:853774-855288	Polyporales
					scaffold_15:846535-848049	
	2383 (PcMnP02_ATCC24725)	PeroxiBase	Pc_MnP02_ATCC24725	MnP	scaffold_5:506005-507526	Polyporales
	2385 (PcMnP03)	PeroxiBase	Pc_MnP03	MnP	U70998	Polyporales
	2382 (PcMnP04)	PeroxiBase	Pc_MnP04	MnP	U10306^‡^	Polyporales
	4636^†^	JGI	Pc_pMnP	MnP	scaffold_7:1395073-1396542	Polyporales
*Phanerochaete sordida*	2387 (PsoMnP01	PeroxiBase	Pso_MnP01	MnP	AB078604	Polyporales
	2388 (PsoMnP02)	PeroxiBase	Pso_MnP02	MnP	AB078605	Polyporales
	2390 (PsoMnP03)	PeroxiBase	Pso_MnP03	MnP	AB078606	Polyporales
*Phlebia radiata*	2401 (PrLiP01)	PeroxiBase	Pr_LiP01	LiP	AY743218	Polyporales
	2297 (PrLiP03)	PeroxiBase	Pr_LiP03	LiP	AY749105	Polyporales
	2400 (PrLiP04)	PeroxiBase	Pr_LiP04	LiP	AY745250	Polyporales
	2296 (PrMnP02)	PeroxiBase	Pr_MnP02	MnP	AJ566199	Polyporales
	2294 (PrCII03)	PeroxiBase	Pr_MnP	MnP	AJ566200	Polyporales
*Pleurotus eryngii*	2301 (PerVP01)	PeroxiBase	Per_VP01	VP	AF007223	Agaricales
	2299 (PerVP02)	PeroxiBase	Per_VP02	VP	AF007224	Agaricales
	2305 (PerVP03)	PeroxiBase	Per_VP03	VP	DQ056374.1	Agaricales
	2302 (PerVP04)	PeroxiBase	Per_VP04	VP	AF175710	Agaricales
*Pleurotus ostreatus*	2391 (PoMnP03)	PeroxiBase	Po_MnP03	MnP	AB016519	Agaricales
	2393 (PoVP01)	PeroxiBase	Po_VP01	VP	U21878	Agaricales
	2392 (PoVP02)	PeroxiBase	Po_VP02	VP	AJ243977	Agaricales
	3871 (PoVP04)^*^	PeroxiBase	Po_VP04	VP	scaffold_6:1045462-1047393	Agaricales
	168144^†^	JGI	Po_pMnP1	MnP	scaffold_5:1707879-1709777	Agaricales
	156366^†^	JGI	Po_pMnP2	MnP	scaffold_4:208389-210297	Agaricales
	153232^†^	JGI	Po_pMnP3	MnP	scaffold_1:2996351-2998068	Agaricales
	29594^†^	JGI	Po_pCII1	CII	scaffold_5:779433-781121	Agaricales
*Pleurotus pulmonarius*	3861 (PpulMnP05)	PeroxiBase	Ppul_MnP05	MnP	AY836676^‡^	Agaricales
*Pleurotus sapidus*	2306 (PsaVP01)	PeroxiBase	Psa_VP01	VP	AM039632^‡^	Agaricales
*Postia placenta*	6737 (PplCII)	PeroxiBase	Pp1_CII	CII	scaffold_58:298384-299825	Polyporales
*Spongipellis* sp.	3897 (SPOspMnP01)	PeroxiBase	SPO_spMnP01	MnP	AB244274^‡^	Polyporales
*Trametes cervina*	2311 (TceCII01)	PeroxiBase	Tce_CII	CII	AB237774	Polyporales
*Trametes versicolor*	2420 (TvLiP)	PeroxiBase	Tv_LiP	LiP	M64993	Polyporales
	2421 (TvLiP07)	PeroxiBase	Tv_LiP07	LiP	Z30667	Polyporales
	2344 (TvLiPGIII)	PeroxiBase	Tv_LiPGIII	LiP	Z30666	Polyporales
	2422 (TvCII01)	PeroxiBase	Tv_MnP	MnP	X77154	Polyporales
	3847 (TvCII03)	PeroxiBase	Tv_VP	VP	AJ745080^‡^	Polyporales
	3848 (TvMnP01_KN9522)	PeroxiBase	Tv_MnP01_KN9522	MnP	AY677128^‡^	Polyporales
	2424 (TvCII05_KN9522)	PeroxiBase	Tv_MnP05_KN9522	MnP	AY677131^‡^	Polyporales
	3857 (TvCII02_PRL572)	PeroxiBase	Tv_MnP02_PRL572	MnP	Z30668	Polyporales
	2425 (TvCII06_KN9522)	PeroxiBase	Tv_MnP03_KN9522	MnP	AY677130^‡^	Polyporales
	2423 (TvCII04_PRL572)	PeroxiBase	Tv_MnP04_PRL572	MnP	Z54279	Polyporales
*Antrodia cinnamomea*	6858 (AciCcP)	PeroxiBase	Aci_CcP	CcP		Polyporales
*Coprinopsis cinerea*	2895 (CcinCcP01)	PeroxiBase	Cc_CcP01	CcP		Agaricales
*Malassezia globosa*	6391 (MaglCcP)	PeroxiBase	Magl_CcP	CcP		Malasseziales
*Phanerochaete chrysosporium*	2821 (PcCcP)	PeroxiBase	Pc_CcP	CcP		Polyporales
*Postia placenta*	6741 (Pp1CcP)	PeroxiBase	Pp1_CcP	CcP		Polyporales
*Ustilago maydis*	2356 (UmCcP01)	PeroxiBase	Um_CcP01	CcP		Ustilaginales

Notes: * Partial amino acid sequences in PeroxiBase, complemented in JGI.

† Obtained from genome and EST database in JGI using tBlastN, and thus marked with a lowercase ‘p’ represented putative.

‡ Only coding sequences were retrieved.

§ Two very similar copies of genome sequences exist in JGI, which may be from a recent duplication, so only one amino acid sequence was used in constructing phylogenetic tree to avoid redundancy.

All other available whole-genome sequences in JGI and NCBI from 11 species/variant species, which represent six basidiomycetous orders (Table 2), were also screened using tBlastN, but no positive hits according to our filtration criterion (see *Data mining* for details) were retrieved, indicating that these species perhaps harbor no LPs.

**Table 2.** Species without LPs and their taxonomic position.

Species	Order	Species	Order
*Cryptococcus bacillisporus*	Tremellales	*Moniliophthora perniciosa*	Agaricales
*Cryptococcus neoformans* var. *grubii*	Tremellales	*Puccinia graminis* f.sp. *tritici*	Pucciniales
*Cryptococcus neoformans* var. *neoformans*	Tremellales	*Schizophyllum commune*	Agaricales
*Malassezia globosa*	Malasseziales	*Sporobolomyces roseus*	Sporidiobolales
*Malassezia restricta*	Malasseziales	*Ustilago maydis*	Ustilaginales
*Melampsora laricis-populina*	Pucciniales		

### Phylogenetic relationship of LPs

**Figure 2.**
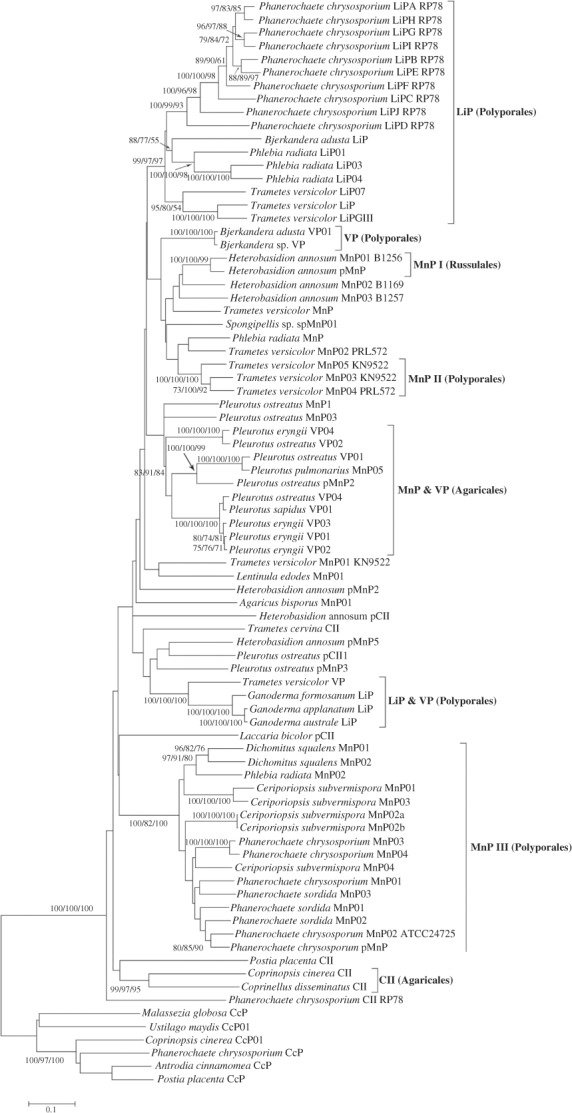
Phylogenetic tree for 76 LPs and 6 CcPs of amino acid sequences without SPs. Topology is from NJ method, while bootstrap values (not less than 50%) from NJ, ML, and MP methods are indicated as percentages at the nodes. Groups LiP, VP, MnP I, MnP II, MnP and VP, LiP and VP, MnP III, and CII are delineated by vertical brackets at the right; meanwhile, the orders, which the LPs within each group belong to, are included in the brackets behind the group names. Six CcPs are set as outgroups.

In the alignment of data set with SPs, 507 characters with 376 informative ones exist. NJ, ML, and MP trees (Supplementary Figure S1) were constructed. After that, all SPs (in Supplementary File 2) were discarded, and the new alignment of the data set without SPs, including 356 informative characters of 492 total characters, was used to construct NJ, ML, and MP trees (Figure 2). Both the two data sets produced congruent phylogenetic topologies; however, for the high similarity of SPs among various LPs, the trees based on the data sets without SPs were more robust than those based on the data sets with SPs and thus were presented (Figure 2). Eight groups could be recognized and were designated according to the LP types in them (Figure 2). Among the eight groups, six groups, namely, LiP, MnP I, MnP II, VP, LiP and VP, and CII, were strongly supported (bootstrap values >90%) in all of the three tree-constructed methods; groups MnP III and MnP and VP well clustered together in all of the three methods (bootstrap values > 80%) and were with high bootstrap values > 90% in at least one method, so we treat them as groups. With regard to *Phelebia radiata* MnP and *Trametes versicolor* MnP02 PRL572, and *Heterobasidion annosum* pMnP5 and *Pleurotus ostreatus* pCII1, they were strongly supported in the NJ method, but they did not cluster well in other two methods (bootstrap values < 50% if cluster), so we treat them as separate LPs.

Six groups contained only a single type of LPs and were found only in a single fungal order as follows: MnP I in Russulales, MnP II in Polyporales, LiP in Polyporales, VP in Polyporales, MnP III in Polyporales, and CII in Agaricales, while two groups had two types of LPs: MnP and VP found in Agaricales, and LiP and VP in Polyporales. It is notable that the LPs grouped neither entirely according to the LP types nor the fungal phylogeny of their original species. Even in the species from the same order, one type of LPs could have different origins. However, no LPs from the species of different orders grouped together.

### Comparison of gene structures versus amino acid sequence alignments

Gene structure analysis was performed in order to deeply probe the relationship among LPs. Certain minor variations in intron positions were accepted, since that may be caused by indel or intron sliding (Stoltzfus et al. 1997). However, the predicted gene structure of Pc_LiPH_RP78 differed from the previous study (Ritch and Gold 1992). It would be corrected if the last four nucleotides of the fourth exon and all four nucleotides of the fifth exon were transferred to introns, and the last eight nucleotides of the fifth intron were transferred to exons. However, the above adjustments would produce a noncanonical 3′-splicing site. This abnormality may stem from errors in large-scale automated sequencing also found in other similar studies (Kawaguchi et al. 2007; Zhou et al. 2008), or it may be real as a similar noncanonical 3′-splicing site has also been identified in other *lp* introns. In either case, there is adequate information to suggest that the previously predicted gene structure of Pc_LiPH_RP78 was not correct and it was consequently adjusted as described above and utilized in subsequent analyses.

**Figure 3.**
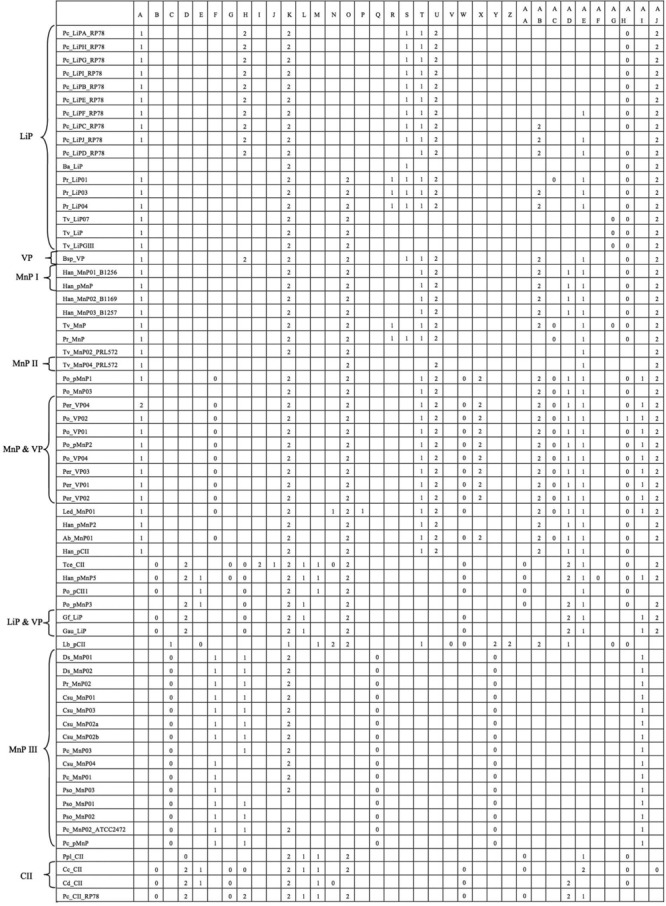
Comparison of the intron position and phase of *lps* based on amino acid sequence alignment. Each intron is named from A to AJ according to its homologue, and its phase is represented by 0, 1, or 2. The LPs from the same groups were assembled together to visualize the comparison clearly, and their code names are the same as those in Table 1.

A total of 36 intron positions were identified and named from A to AJ (Figure 3). Several LPs were excluded from the comparison, because their gene structures were unavailable (Table 1). This produced that only a single LP existed in groups VP and MnP II in the comparison, and thus it was unable to compare *lp* gene structures in these two groups, whereas it would be possible to do these in other groups, especially in groups LiP, MnP and VP, and MnP III with 17, 8 and 15 LPs, respectively, and thus obtain some general information. Whole *lp* gene structures were not highly conserved and only one intron, K with a nearly identical phase 2, appeared in all groups. However, most introns coexisted in several groups, such as introns A in groups LiP, VP, MnP I, MnP II and MnP and VP, O in groups LiP, VP, MnP I, MnP II, MnP and VP, LiP and VP and CII, T in groups LiP, VP, MnP I and MnP and VP, and so on. Five introns appeared only in certain *lp* genes: introns J in Tce_CII, P in Led_MnP01, V and Z in Lb_pCII, and AF in Han_pMnP5. Remarkably, the introns within the same phylogenetic group, especially those simultaneously in the same species or even the same genus, were fairly conserved in both position and phase. For example, in group LiP, introns H, S, T, U, and AJ, intron R, and intron AG were exactly identical in each LP of *P. chrysosporium, P. radiata,* and *T. versicolor,* respectively; in groups MnP I, MnP and VP, and LiP and VP, each intron of LPs appeared in the same positions with identical phases; in group MnP III, each LPs possessed the same four introns, C, Q, Y, and AI.

### Selective pressure acting on LPs

The selective pressure on all LPs (see Supplementary File 3 for their coding sequences) was evaluated using three pairs of site models. M8 indicated positive selection with ω = 2.38, and many positively selected sites were identified by NEB, most of which were at the 99% level (*P* > 99%; Supplementary Table S1), but the LRT statistic of M7 vs. M8 rejected the hypothesis (Table 3). No unambiguous positive selection was thus found in the whole LP family. Subsequently, each of the eight LP groups was analyzed separately using the same three site models. Positive selection in groups MnP I, MnP III, and CII was ensured by M0 vs. M3 (ω = 1.21, *P* < 0.01) and M7 vs. M8 (ω = 1.49, *P* < 0.025), M0 vs. M3 (ω = 1.56, *P* < 0.01), and M0 vs. M3 (ω = 19.89, *P* < 0.01), respectively, while in the other groups, positive selection was rejected either by an ω value not more than 1, or by a *P* value not less than 0.05 in LRT statistics, following the rules of Yang (2000).

**Table 3.** LRT statistics and *P* value.

	M0 vs. M3 (d.f. = 4)			M1a vs. M2a (d.f. = 2)			M7 vs. M8 (d.f. = 2)		
Group	2Δℓ		*P* value	2Δℓ		*P* value	2Δℓ		*P* value
All sequences		–			–		–11044.875094		–
LiP		–			–				
VP	6.663864		>0.10	1.475886		>0.10	2.073648		>0.10
MnP I	24.743772		<0.01	6.744184		<0.05	6.904412		<0.05
MnP II		–			–				
MnP & VP		–			–				
LiP & VP		–			–				
MnP III	220.201258		<0.01		–				
CII	56.385656		<0.01	1.777202		>0.10	2.001702		>0.10

## Discussion

In this study, we assembled the largest collection of LPs to date, explored their phylogeny, and examined the mechanisms mainly caused the significant differences between white-rot and brown-rot fungi in degrading lignin.

The most notable feature of the phylogenetic analysis was their topologic structures. The LPs of different types and/or from different species often clustered in the same group (MnP and VP and LiP and VP) with high bootstrap values, and vice versa (MnP I-III). This might be caused by the established standard for the phylogenetic topologic structure based on the information of overall amino acid sequences is different from the phylogeny of the LP types based solely on several key residues involved in substrate oxidation. It is the topologic structure rather than the LP types that reflects the bona fide phylogenetic relationship. Given the LPs in each group evolved independently and thus had their own unique ancestors, we could get some information on the origin of LPs from the phylogenetic relationship: (1) the same types of LPs might originate from different ancestors independently, such as MnP from groups MnP I, MnP II, MnP and VP and MnP III, LiP from groups LiP and LiP and VP, and VP from groups VP and MnP and VP. (2) Even in the same species, different types of LPs might have not only one origin. For example, MnPs, LiPs and VP of *Trametes versicolor* (L.) Lloyd fell into groups MnP II, LiP and LiP & VP, respectively, but not assembled together. So did LiPs and MnPs of *P. chrysosporium*, LiPs and MnP of *Phlebia radiata* Fr., and LiP and VP of *Bjerkandera adusta* (Willd.) P. Karst. (3) Even the same type of LPs from one species might not cluster in the same group. Four MnPs from *Heterobasidion annosum* Fr. (Bref.) and two from *T. versicolor* were scattered in the trees rather than clustered together within the corresponding groups MnP I and MnP II, respectively. This indicated that these MnPs either originated from different ancestors or experienced some relatively dramatic changes in amino acid sequences. (4) The LPs within the same groups only existed in an identical order, although LPs from the same order could have multiple origins. This suggested that the origins of LPs were independent at the level of orders. In fact, during the screening of fungal genome sequences, we found that nine taxa from other five orders outside Russulales, Agaricales, and Polyporales (Table 2) did not have any positive hits. It could be postulated that not all basidiomycetous orders had LPs, in despite that the genome of many basidiomycetous species are not sequenced at all.

According to our searches of all available fungus and LP data, CII and MnP exist in the orders of Russulales, Agaricales and Polyporales, VP in Agaricales and Polyporales, and LiP only in the Polyporales (Table 1). The regularity of the distribution of LPs in the three orders was substantial, and thus it seems that with the aid of other evidence we could presume the evolutionary order of various LP types. For example, CII and MnP exist in all three orders and are more widespread than other types (Orth et al. 1993), suggesting that they were the earliest extant LP types. After them, VP, existing in two orders, appeared, and then LiP emerged in Polyporales. This evolutionary order met the need of white-rot fungi to degrade the heterogeneous and recalcitrant lignin polymer. It could be postulated that in response to the appearance of lignin with high redox potential, the ancestor of LPs evolved from CcPs through the modifications of some key amino acid residues (Passardi, Bakalovic, et al. 2007). CII without any obvious lignin-degrading activity and MnP degrading only phenolic lignin units were the primary groups of enzymes to evolve. Following them, VP and LiP with a capacity to degrade nonphenolic structures, the most lignin units (Boerjan et al. 2003), emerged, and thereafter lignin could be degraded completely and efficiently. This conjecture is mostly based on the evolutionary order of the three basidiomycetous orders: from Russulales to Agaricales, and then to Polyporales. However, there is still ambiguity on the evolutionary order of the three basidiomycetous orders (Hibbett et al. 2007), when various data sets and phylogenetic methods were used. So making sure the evolutionary order of the three orders would dramatically help us to elucidate the origins of various LP types.

Besides the similarities of the amino acid sequences (directly reflected by the topologies of phylogenetic trees), *lp* gene structures were also used to show the relationships among various LPs. The gene structures were, more or less, conserved within each group relative to those among different groups, suggesting that the LPs within the same groups did have their own common ancestors, despite that they had different enzyme types and were even from different species. The ancestors within each group either yielded a series of LPs with a single type or diversified into different LP types in various fungal species.

It seems easily to understand that the LPs from a common ancestor have an identical enzyme type, whereas more evidence is needed to explain how to produce not only one LP type from a common ancestor. The topologies of groups MnP and VP and LiP and VP showed that the enzyme types could be transformed between MnP and VP, and LiP and VP, respectively. Furthermore, the transformation has been successfully carried out *in vitro* by site-directed mutagenesis (Timofeevski et al. 1999; Mester and Tien 2001). Besides that, as previously mentioned, the LP lies on several residues involved in the process of oxidization. Therefore, selective pressures on LPs were analyzed to detect the dynamic of the transformations, namely, whether positive selection acted on LPs.

The ω value and related parameters of LPs were estimated with PAML 4.2 using the CODEML program. Positive selection was rejected in the whole LPs, but was doubtlessly ensured in the groups MnP I, MnP III, and CII under the used site models: M0 vs. M3 and M7 vs. M8, M0 vs. M3, and M0 vs. M3, respectively. This indicated that the LPs in these three groups were with a potential bias to transfer to other LP types. The residues with the bias to mutation (Supplementary Table S1) were worth investigating further to show the functions of them in the oxidization reactions. Instead of tryptophan residue, tyrosine residue (Y181) was found a catalytic residue in the LiP of *Trametes cervina* (Schwein.) Bres. (Miki, Calvino, et al. 2011). Moreover, the chemically modified study also indicated that tyrosine could be considered as an effective catalytic site in the LiP of *P. chrysosporium* (Miki, Ichinose, et al. 2011). Therefore, positive selection also has a potential role in producing more unknown types of LPs. With regard to MnP type, only transferring to VP type was identified from the phylogenetic topology (the MnP and VP group). Through this transformation, LPs got a potential capacity to degrading high redox lignin structures. However, it is hard to exclude the possibility that MnP type could transfer to LiP or CII types. Given that positive selection did not act on all LPs, but only on three of eight groups, adaptive evolution of LPs might only have occurred in a small fraction of fungal species that occupied a special niche. Large-scale ecological study will reveal why the positive selection occurred in these rather than in other species, and what was the driving force for the positive selection. Besides the above calculated evidence on the transformation of LP types, CIIs also occured in a few brown-rot fungi, such as Ppl_CII predicted in the *P. placenta* genome. Moreover, recently a functional LP closely related to VP was identified in a well-known brown-rot fungus *Antrodia cinnamomea* T.T. Chang and W.N. Chou (Huang et al. 2009). These phonomena indicate that the transformations of LP types could change the lignin degrading ability of wood-decaying fungi and even the rot types of them. In other words, if LP types in brown-rot fungi were transformed to either MnP, LiP or VP from CII, the brown-rot fungi might also be changed to white-rot fungi, and likewise if all LPs in white-rot fungi were transformed to CII type, the white-rot fungi might be brown-rot fungi as well.

Two other species of the order Agaricales, *Moniliophthora perniciosa* (Stahel) Aime and Phillips-Mora and *Schizophyllum commune* Fr., lacked any type of LPs (Table 2). This might be considered normal for the former as it is a nonwood-decaying fungus, but is surprising in the latter case, as *S. commune* Fr. is a ubiquitous white-rot fungus with a worldwide distribution and its genome sequences detected in this study are quite complete (up to ∼8.29× coverage). The presence of laccases, the second type of lignin-degrading enzymes, has been shown in *S. commune* (Hatamoto et al. 1999), suggesting that it contains a different system for lignin degradation and causes white-rot compared to the LP system in some other white-rot fungi. Taking *P. chrysosporium* with LPs and without laccases (Martinez et al. 2004) into consideration together, it seems that there are two distinct lignin-degrading systems, which convergently evolved in function, and white-rot fungi obtained either one or both of them. Laccases also exist in insects, plants, and bacteria besides fungi, and they have multiple functions in different kinds of species (Nitta et al. 2002; Claus 2003; Arakane et al. 2005); however, recent evolutionary studies only focused on laccases from fungi (Valderrama et al. 2003), and even Hoegger et al. (2006) including wider range of taxa did not elucidate the relationship among laccases from the four kinds of species. Therefore, phylogenetic studies, reflecting to the origin and evolution of fungal laccases, were needed to explore whether the phylogeny and lignin-degrading ability of fungal laccases entirely differed from those of LPs.

The evolutionary pattern of multigene families has two models: concerted evolution and birth-and-death evolution as defined by Nei and Rooney (2005). As seen in the above discussion, sometimes LPs from the same species were not closer to each other than to those from other species in phylogeny, and there are at least twice widespread duplication events in their evolutionary history. One brought the ancestors of each group from the most primitive ancestor, and the other diversified the members of each group. Among the various LP types, besides MnP, LiP, and VP, there was also a potentially nonfunctional CII and perhaps also some other types derived from the above gene duplication events. Thus, the *lp* gene family might be subjected to the birth-and-death evolution just as the Class-I of the plant peroxidases superfamily (Zámocký 2004). Similarly, in groups MnP and VP and MnP III, the LP clusters did not follow strictly the phylogeny of their original species. This indicated that the gene duplication events probably occurred before the divergence of these fungal species or genera, and the duplicated LPs were passed down to newborn species or genera, respectively, after the divergence. The conservation of gene structures in each of the four groups also supports the theory that the LPs in these four groups were subjected to birth-and-death evolution. Conversely, group LiP contained 17 LiPs from four species, or say genera, belonging to the same order, Polyporales, and the LiPs from the same species/genus clustered together with strong supports. Furthermore, except for the introns shared in all genes of this group, there were also some species- or genus-specific introns. Thus the multiple LiP copies in one species or genus might be derived from an intraspecies/intragenus gene duplication event, which means that the LiPs in group LiP were subjected to concerted evolution. With regard to the groups MnP I, MnP II, VP, LiP and VP, and CII, the number of LPs in each one was too low to exactly infer the situation of gene duplication events, much less to identify the evolutionary patterns in these groups. We can, however, conclude that the *lp* gene family was subjected to the mixed process of both concerted and birth-and-death evolution.

In summary, this study may provide essential evidence for elucidating the evolutionary dynamics of LPs and thus gaining insights into the alternation of white-rot and brown-rot fungi in their common lineage. LPs evolved in the mixed models of concerted and birth-and-death manners. They originated multi-times at the level of orders independently, and thus they did not exist in all basidiomycetous orders, but only in several (three according to current study) orders. After the duplication events of initial LPs, which provided the raw materials for mutation events, various LP types might occur through mutations of several key residues driven by positive selection. This dynamics still acted on LPs from a small fraction of species, and might change LP types and even alter the rot types of wood-decaying fungi. It is still hard to conclude the evolutionary orders of various LP types. However, as the strict regularity of the distribution of LP types in Russulales, Agaricales, and Polyporales, it could be postulated as soon as we knew the evolutionary orders of the three basidiomycetous orders. These discoveries explain why white-rot and brown-rot fungi alternate in their lineage and will also provide a foundation for further research on the application of LPs in degrading persistent organic pollutants.

## Funding

The research was financed by the National Natural Science Foundation of China [Project no. 31200015] and the Youth Fund for Creative Research Groups, Institute of Applied Ecology, Chinese Academy of Sciences.

## Supplemental data

Supplemental data for this article can be accessed here.
